# First Principles of Medicine

**Published:** 1851-03

**Authors:** 


*First Principles of Medicine.* By ARCHIBALD BILLING, M. D., A. M., F. R. S., formerly Professor of Clinical Medicine and Physician of the London Hospital. *Second American, from the revised and improved fifth London edition.* 8vo, pp. 246. Philadelphia, Lea & Blanchard, 1851.

The merit of this little book may be considered pretty well established, after five London and two American editions, within the space of twenty years. And it is certainly not surprising that so spirited a glance at general pathology, combined with an excellent therapeutic analysis, should have taken a hold of the profession. It hardly, however, deserves place as a systematic treatise on the 'Principles of Medicine': although Dr. Billing flatters himself that, in his later editions, he has shaped his work to this end. We suspect that it will continue, as he first found it to be, "more pleasing to fully educated men and men of experience, than to the tyro." To the reader of the former class, however, who has already studied the principles of medicine, a suggestive, original work like this, stored with practical facts, must always be of great value and interest.

A subject of general criticism from the first edition of this book, has been the absence of a table of contents, and of the usual division into chapters and sections. These useful adjuvants the author, we regret to say, persists in discarding, through all his editions, with the exception of an index which he has added to the fifth, in deference to the wishes of others. Perhaps the omission is to some extent a key to the character of the book—in the facility which it affords to the discursive style in which the author indulges.

One of Dr. Billing's points in his opening or theoretical observations, is the cause of the *first* sound of the heart, which he affirms to depend, like the second, on valvular contraction. Considerable space is devoted to the discussion of this topic, but we believe that Dr. B. stands nearly alone in denying the agency of muscular action in producing the first sound of the heart. The received opinion is, undoubtedly, that which ascribes this sound chiefly to muscular contraction.

Much the most valuable portion of the 'Principles,' is devoted to the examination of therapeutic agents. They are noticed under the division into stimulants, sedatives, narcotics, and tonics; and the author here goes far toward fulfilling the anticipations held out in the preface to one his editions, "that it will be found to contain the essentials of the treatment of disease." If not a complete 'guide to clinical practice,' the book undoubtedly presents a very sound and original train of reflection on the subject—extremely well condensed—which cannot be read without much profit and interest.

